# A Practical Guide to the Roles of Procalcitonin Measurement in Patients with Acute Pancreatitis

**DOI:** 10.3390/jcm15031153

**Published:** 2026-02-02

**Authors:** Ajith K. Siriwardena

**Affiliations:** 1 Regional Hepato-Pancreato-Biliary Unit, Manchester Royal Infirmary, Manchester M13 9WL, UK; a.siriwardena@btinternet.com; 2 Faculty of Biology, Medicine and Health, The University of Manchester, Oxford Road, Manchester M13 9PL, UK

**Keywords:** acute pancreatitis, procalcitonin, infection, pancreatic necrosis, randomized trial

## Abstract

**Background/Objectives:** This review is a concise summary of the current roles and indications for procalcitonin measurement in the management of patients with acute pancreatitis. **Methods**: The National Library of Medicine’s PubMed database for the period 1 January 2000 to 27 November 2025 was interrogated using the keywords “procalcitonin” and “acute pancreatitis”. Articles on gut dysbiosis in acute pancreatitis, procalcitonin and its role as a predictor of disease severity, a marker of pancreatic necrosis and a guide to antibiotic therapy in acute pancreatitis were retrieved. **Results**: Persistently elevated procalcitonin levels are indicators of disease severity and necrosis in acute pancreatitis. In the setting of acute pancreatitis with raised inflammatory markers, a procalcitonin level >1 ng/mL has evidentiary proof for use as an indicator for starting antibiotic therapy. **Conclusions**: In current practice, in patients with acute pancreatitis and raised inflammatory markers but without positive microbiological cultures, a procalcitonin level >1 ng/mL can be used as an indicator for starting antibiotic therapy.

## 1. Introduction

Acute pancreatitis is inflammation of the pancreas. In the United Kingdom, the estimated incidence is 56 cases per 100,000 [1]. There is evidence globally of an increase in incidence [2]. A typical clinical presentation is acute onset of abdominal pain and vomiting (although variant presentations are common). Most episodes follow a clinically mild course and require supportive therapy only; however, a proportion—up to one-third—will have a more severe clinical illness characterized by organ failure [3]. In these patients, there is often underlying pancreatic necrosis [3]. The treatment remains supportive with emphasis on fluid replacement, analgesia, organ support and nutrition [1]. The role of antibiotic therapy in acute pancreatitis has been the subject of much attention with a recent systematic review of twenty-one trials including 1383 patients showing that antibiotic prophylaxis was not associated with any difference in the incidence of infection of pancreatic necrosis or episode-related mortality [although there was evidence of a reduction in extra-pancreatic infection with a decreased risk of sepsis (RR 0.43; 95% CI 0.25–0.73) and urinary tract infections (RR 0.46; 95% CI 0.25–0.86)] [4]. Trial sequential analysis (a method of meta-analysis used to weight Type I and II errors and estimate when the effect is unlikely to be altered by further trials) concluded that no further trials of antibiotic prophylaxis in acute pancreatitis are required [4,5].

The recently published International Association of Pancreatology revised guidelines for the treatment of acute pancreatitis do not recommend antibiotic prophylaxis in patients with acute pancreatitis [6]. Antibiotics do however have important roles in the treatment of patients with proven infection in acute pancreatitis [6].

A practical clinical problem for those treating patients with acute pancreatitis is decision making regarding whether or not to use antibiotics in the scenario of a patient with features of clinical infection (such as fever, tachycardia and flushing) together with raised inflammatory markers (leukocyte count and C-reactive protein) as the systemic inflammatory response of this disease closely mimics infection [7]. In this setting, procalcitonin measurement may help to guide therapy.

Procalcitonin is currently referred to as a hormokine as it shares characteristics of hormones and cytokines and has an important role in maintaining vascular endothelial tone in response to bacterial infection [8]. Procalcitonin concentrations increase rapidly in response to a pro-inflammatory stimulus of bacterial origin and decrease after successful treatment [8]. Procalcitonin is more sensitive than clinical assessment and routine laboratory markers of sepsis (such as leukocyte count and C-reactive protein) in detecting pancreatic infection [9].

The PROCAP randomized control trial demonstrated that the use of a procalcitonin-based algorithm to guide antibiotic therapy in acute pancreatitis resulted in a reduction in antibiotic use without an increase in overall or infective complications [10]. The aim of this review is to provide a concise, practical overview of the current roles of procalcitonin measurement in patients with acute pancreatitis.

## 2. Methods

### 2.1. Design

This article is a concise review of the roles of procalcitonin measurement in patients with acute pancreatitis. A search of the National Library of Medicine’s PubMed database for the period 1 January 2000 to 27 November 2025 using the keywords “procalcitonin” and “acute pancreatitis” yielded 296 results. Of these, 199 were excluded as not relevant, 30 were not related to acute pancreatitis, 8 were letters without published abstracts, 6 were reviews, 2 were not in English and 1 was a guideline. In total, 50 original peer-reviewed articles relating to procalcitonin measurement in acute pancreatitis comprise the data sources for this review. Further searches using the keyword “pancreatic necrosis” in addition to “procalcitonin” and searches of OVID and the Web of Science databases did not yield further articles.

### 2.2. Sections of Review

#### 2.2.1. Gut Dysbiosis in Acute Pancreatitis

The role of the gut in mediating inflammation in acute pancreatitis is reviewed together with a discussion of the role of gut barrier function and other changes during the disease course of acute pancreatitis.

#### 2.2.2. Procalcitonin

The nature of procalcitonin and its role as a marker of infection are overviewed here, including studies that support its role and those that do not.

#### 2.2.3. Procalcitonin as a Predictor of Disease Severity in Acute Pancreatitis

The role of procalcitonin measurement as a predictor of disease severity and outcome in acute pancreatitis is discussed in this section.

#### 2.2.4. Procalcitonin as a Marker of Pancreatic Necrosis

The role of procalcitonin measurement as an indicator of pancreatic necrosis is reviewed in this section.

#### 2.2.5. Procalcitonin Measurement to Guide Antibiotic Therapy in Acute Pancreatitis

This section overviews the PROCAP randomized controlled trial of the use of a procalcitonin measurement algorithm to guide antibiotic therapy in patients with acute pancreatitis. Other trials using procalcitonin measurement to guide antibiotic therapy are also reviewed.

#### 2.2.6. Future Directions

This section will provide an overview of near-future developments in the role of procalcitonin measurement in acute pancreatitis.

### 2.3. Ethics Approval

This review involved no patient contact and did not require prior ethics committee approval.

### 2.4. Protocol Registrations

The study protocol was not registered with any organization prior to publication.

## 3. Results

### 3.1. Gut Dysbiosis in Acute Pancreatitis

Changes in gastrointestinal function are central to both the clinical presentation and the underlying pathophysiology of acute pancreatitis. Clinically, the typical presentation is marked by acute, severe abdominal pain associated with vomiting. In experimental models of acute pancreatitis, there is loss of the normal physiological propulsive action of the proximal small bowel with an absence of migrating myo-electric complexes [11]. This stasis likely contributes to the nausea and vomiting seen clinically. It is also associated with a rapid change in the composition of the upper gastrointestinal microbiome [12]. Bacterial proliferation in the proximal small intestine is also associated with activation of host intestinal brush border inflammatory cells, which contribute to the ongoing inflammatory process [13]. Gastrointestinal blood flow is also altered in the early phases of acute pancreatitis, with splanchnic hypo-perfusion contributing to gut dysfunction [14]. There is good evidence that gastrointestinal permeability is altered in acute pancreatitis, with an increase in absorption of normally non-absorbed large molecular weight sugars having been observed [15]. Intestinal tight barrier function is also compromised, resulting in potential access to the circulation for bacterial-derived toxins [16].

All of these changes occur early in the clinical course of acute pancreatitis and are not regarded as infection. Prolonged small bowel stasis can be seen in critically ill patients with acute pancreatitis and is often labelled “ileus”.

Restoration of intestinal function is a key component of recovery from acute pancreatitis.

### 3.2. Procalcitonin

Procalcitonin is a 116-amino acid peptide produced by the parafollicular C cells of the thyroid and by neuro-endocrine cells in bronchi and intestinal crypts [17]. The gene for procalcitonin is located on the CALC-1 gene on chromosome 11 [18]. Bacterial infections induce a universal increase in CALC-1 gene expression and a release of procalcitonin into the circulation [18]. In a “normal” physiological state, procalcitonin is converted to calcitonin so that the level of procalcitonin in the bloodstream of healthy individuals is below the limit of detection (0.01 μg/L) of clinical assays [17]. During inflammation, procalcitonin is produced mainly by two alternative mechanisms: a direct pathway induced by lipopolysaccharide or other toxic metabolites from microbes and an indirect pathway induced by inflammatory mediators such as interleukin 6 (IL-6) and tumour necrosis factor alpha (TNF-α), resulting in a rapid rise in circulating levels [17]. Critically, procalcitonin levels do not rise in the setting of non-infectious inflammation—as seen in the early phases of acute pancreatitis. Procalcitonin levels fall after successful treatment of bacterial infection [17,18]. As a result, algorithms based on procalcitonin measurement have become accepted in differentiating between bacterial infection and inflammation [19].

#### Procalcitonin Assay

Human Procalcitonin is measured by a solid-phase sandwich ELISA (enzyme-linked immunosorbent assay [17]). Samples, standards, or controls are added to wells and bind to an immobilized (capture) antibody. The sandwich is formed by the addition of the second (detector) antibody, and a substrate solution is added that reacts with the enzyme–antibody–target complex to produce a measurable signal. The intensity of this signal is directly proportional to the concentration of the target present in the original specimen. The assay is utilizable in clinical practice with a rapid turnaround time for individual samples without the delays inherent in batch assay and sampling [17].

### 3.3. Procalcitonin as a Predictor of Disease Severity in Acute Pancreatitis

As the initial clinical presentation in acute pancreatitis may not correlate with the subsequent disease course, many methods to predict severity have been assessed. Multiple biochemical and clinical factor prediction scores, such as the APACHE II and BISAP scores, have been developed and validated [20,21]. Individual biomarker tests have also been proposed. For example, an elevated C-reactive protein at 48 h into the episode is predictive of the presence of pancreatic necrosis [22]. Interleukin-6 has also been used to predict disease severity [23].

Mofidi and colleagues undertook a meta-analysis in 2009 of studies evaluating procalcitonin as a predictor of the development of severe acute pancreatitis. Twelve studies, including 826 patients, were included [24]. All were prospective studies in which severe acute pancreatitis was compared to mild or sterile necrosis to the infected form, with heterogeneity in both sampling intervals and, crucially, in the method of assay of procalcitonin. Notwithstanding these heterogeneities, the pooled sensitivity and specificity of procalcitonin for the prediction of severe acute pancreatitis were 0.72 (95% CI = 0.64–0.77) and 0.86 (95% CI 0.83–0.89), respectively, with an overall area under the curve on receiver operator curve analysis of 0.865 [24]. The authors observed a significant degree of heterogeneity in the studies within their analysis, with a Q statistic of 28.56 (*p* < 0.01). When their analysis was restricted to the eight studies, which used a procalcitonin cut-off for prediction of infection of 0.5 ng/mL, there was a modest increase in sensitivity and specificity, together with an increase in AUC to 0.88 and a reduction in Q [24].

Procalcitonin measurement can be combined with other indices of severity to create a nomogram for the prediction of adverse outcomes in acute pancreatitis [25].

A meta-analysis of the diagnostic value of procalcitonin in patients with severe acute pancreatitis based on 18 studies including 1764 patients indicated that procalcitonin has good sensitivity and diagnostic accuracy for the prediction of severe acute pancreatitis. The area under the curve was 0.89 (95% CI: 0.86 to 0.92) [26].

Table 1 is a summary of selected studies using procalcitonin measurement as a predictor of disease severity. Six studies reporting on 1837 patients with acute pancreatitis, two of which were undertaken prospectively, showed that elevated procalcitonin correlated with disease severity [9,27,28,29,30,31]. It should be noted that sampling and study methodology were heterogeneous.

The current IAP/APA guidelines recommend that systemic inflammatory response syndrome (SIRS) at admission and persistent SIRS at 48 h from the onset of abdominal pain, either alone or in combination with a high C-reactive protein (CRP) or Interleukin-6 (IL-6), should be used to predict severe acute pancreatitis (strong recommendation; moderate quality evidence) [6]. Procalcitonin measurement, either alone or as part of a marker panel or nomogram, is not recommended for predicting episode severity.

### 3.4. Procalcitonin as a Marker of Pancreatic Necrosis

Mofidi and colleagues’ meta-analysis also assessed the value of procalcitonin in predicting the development of infected pancreatic necrosis [24]. Their study assessed the sensitivity, specificity, positive predictive value and negative predictive value of procalcitonin for predicting the development of infected pancreatic necrosis (defined as either positive fine needle aspiration culture or operative findings). Seven studies evaluated the value of procalcitonin as a predictor of infected necrosis. The pooled sensitivity and specificity of procalcitonin for the prediction of infected necrosis were 0.80 (95% CI 0.7–0.87) and 0.91 (95% CI 0.87–0.94), respectively [24]. The area under the curve (AUC) of a receiver operator curve was 0.91. The authors concluded that although the exact place of procalcitonin in the management of patients with acute pancreatitis remains to be defined, serum procalcitonin as an early marker of the development of infected necrosis can be a useful adjunct to conventional severity stratification and a guide to the progression of the disease.

The passage of time does not diminish the quality of this important meta-analysis or its carefully phrased conclusion. Management of ongoing acute pancreatitis, especially in patients with pancreatic necrosis, has evolved since the publication of the Mofidi meta-analysis. Supportive care remains the mainstay of management, but randomized trial evidence demonstrates that early intervention is not beneficial, and for those with localized necrotic collections, a step-up approach with delayed intervention achieves better outcomes [32]. A systematic review and meta-analysis of 18 studies (2 randomized controlled trials and 16 observational studies) comprising 2392 patients with symptomatic post-inflammatory acute necrotic pancreatic collections demonstrated that early intervention was associated with increased overall mortality (OR 1.73, 95% CI 1.34 to 2.22, *p* < 0.001, I2 = 4%; 18 studies) and a greater need for open surgery (OR 1.77, 95% CI 1.23 to 2.56, *p* = 0.002, I2 = 39%; 11 studies) compared to delayed intervention. However, most studies were assessed to have a high risk of bias [33].

Table 2 is a selection of studies that report the utility of an elevated procalcitonin for the prediction of pancreatic necrosis [9,28,34,35,36]. These five studies report on a series of 556 patients. There is no agreed cut-off value of procalcitonin for prediction of necrosis, although Choudhuri reported persistently elevated procalcitonin levels in non-survivors with severe acute pancreatitis [34]. Alberti reported that elevated levels also helped to detect other complications such as cholangitis [28].

Prediction of pancreatic necrosis is not specifically assessed in the current IAP/APA guidelines, but the available evidence does not provide strong support for serial procalcitonin measurement to monitor the evolution of acute pancreatitis.

### 3.5. Procalcitonin Measurement to Guide Antibiotic Therapy in Acute Pancreatitis

Procalcitonin measurement was first evaluated as a guide for antibiotic cessation in patients with acute pancreatitis in a randomized trial by Qu and colleagues [37]. In their study, patients in the control group received antibiotics for 14 days. As this is a substantial deviation from worldwide antibiotic use in patients with acute pancreatitis, it is not possible to extrapolate from the findings of Qu and colleagues’ study.

The PROCAP randomized trial was a single-centre, patient-blind randomized controlled trial of the use of a procalcitonin measurement-based algorithm to guide empiric antibiotic use in patients with acute pancreatitis [10]. The algorithm is seen in Table 3. Adult patients were enrolled if they met two of the three diagnostic criteria for acute pancreatitis defined in the 2012 update of the Atlanta consensus [3]. Patients were randomly allocated on a 1:1 basis to either procalcitonin-guided antibiotic use or standard care. To ensure a balanced trial, two stratifications were used. First, patients were stratified by mild or moderate/severe disease and second, by whether they were direct admissions to the hospital from the emergency room or tertiary transfers from other hospitals. In the intervention arm, per protocol, procalcitonin testing was conducted on days 0, 4, 7, and weekly thereafter, using the Elecys BRAHMS fully automated procalcitonin immunoassay (BRAHMS Assay; Roche Diagnostics, Rotkreutz, Switzerland). The threshold for a positive procalcitonin test for this study was 1·0 ng/mL.

It is important to appreciate that procalcitonin measurement was one of a series of considerations guiding antibiotic use in this trial. Where patients had positive microbiology results or required antibiotic prophylaxis to cover interventional procedures such as laparoscopic cholecystectomy, this was permitted for patients in either arm.

Standard care followed the 2013 International Association of Pancreatology/American Pancreatic Association guidelines for the management of patients with acute pancreatitis (which represented the standard of care at the time of the study) [38]. All aspects of care, with the exception of the use of a procalcitonin algorithm to guide antibiotic use, were the same for patients in both study groups. The primary outcome was antibiotic use (binary endpoint: yes or no) during the index stay. A range of secondary outcomes included incidence of infections, complications and episode-related mortality.

Between 29 July 2018 and 13 November 2020, 369 patients were screened, of whom 109 were excluded and 260 were enrolled and randomly assigned to a treatment group (132 to procalcitonin-guided care and 128 to usual care. Baseline demographics, including age, gender distribution, etiology and disease severity, were similar between groups.

A total of 59 (45%) patients in the procalcitonin-guided care group were prescribed antibiotics compared to 79 (63%) in the usual care group (adjusted risk difference −15.6% [95% CI −27.0 to −4.2]; *p* = 0.0071). The mean number of days of antibiotic use per patient was significantly lower in the procalcitonin-guided care group than in the usual care group.

There was no significant difference between groups in the number of clinical infections per patient. The incidence of clinical infection by pathogen (including isolates of multiresistant bacteria), together with the overall rate of infected pancreatic necrosis, showed no significant difference between groups in terms of the number of hospital-acquired infections per patient. There was also no difference in the use of radiological, endoscopic or surgical intervention between the treatment groups.

The study concluded that procalcitonin-guided care can reduce antibiotic use without increasing infection or harm in patients with acute pancreatitis. The study recommended that procalcitonin-based algorithms to guide antibiotic use should be considered in the care of this group of patients and be incorporated into future guidelines on the management of acute pancreatitis.

### 3.6. Future Directions

The scientific validity of the findings of the PROCAP study has been supported by the results of the large-scale ADAPT-SEPSIS randomized controlled trial [39]. This was a multi-centre, intervention-concealed randomized clinical trial, involving 2760 adults requiring critical care within 24 h of initiating intravenous antibiotics for suspected sepsis and likely to continue antibiotics for at least 72 h within the United Kingdom’s National Health Service. From 1 January 2018 to 5 June 2024, 918 patients were assigned to the daily procalcitonin-guided protocol, 924 to the daily CRP-guided protocol and 918 to standard care.

The results showed that there was a significant reduction in antibiotic duration in those patients in the procalcitonin-guided protocol compared with standard care (mean duration, 10.7 [SD, 7.6] days for standard care and 9.8 [SD, 7.2] days for PCT; mean difference, 0.88 days; 95% CI, 0.19 to 1.58, *p* = 0.01). For all-cause mortality up to 28 days, the daily procalcitonin-guided protocol was non-inferior to standard care, where the noninferiority margin was set at 5.4% (19.4% [170 of 878] of patients receiving standard care; 20.9% [184 of 879] receiving procalcitonin-guided care; absolute difference, 1.57; 95% CI, −2.18 to 5.32; *p* = 0.02). The CRP guided protocol showed no difference from standard care. The authors concluded that care guided by measurement of procalcitonin reduces antibiotic duration safely compared with standard care, but CRP does not [39].

This study provided independent, current and rigorous corroboration of the findings of the PROCAP study.

Future directions in antibiotic stewardship include the incorporation of procalcitonin measurement into a comprehensive antibiotic stewardship protocol.

The Precision use of Antibiotics in Infected NecrOtizing Pancreatitis (PIANO) trial is a multicenter, cluster-randomized, non-inferiority trial in all Dutch hospitals collaborating within the Dutch Pancreatitis Study Group (DPSG). The study plans to include all hospitalized adult patients with necrotizing pancreatitis. A bundle approach based on antibiotic stewardship principles will be compared with current care. The main objective will be to compare current care with the implementation of a structured and multifaceted approach based on antibiotic stewardship principles for patients with necrotizing pancreatitis in terms of mortality, major complications, number of interventions, hospital stay and quality of life [40].

## 4. Discussion

This review has examined the role of procalcitonin measurement in the contemporary management of patients with acute pancreatitis.

The underlying changes in gut structure and function and the role of gut dysbiosis were reviewed, and it was emphasized that these changes in the early phases of acute pancreatitis do not correlate with infection.

The physiology of procalcitonin and procalcitonin measurement-based algorithms was then reviewed. In this regard, procalcitonin measurement is now accepted as helping to guide more accurate antibiotic prescribing and a shorter duration of antibiotic therapy in a range of clinical settings, including neonatal critical care and community-acquired pneumonia [41,42]. In order to present a balanced perspective, it should also be pointed out that in a large study of 1656 patients admitted to hospitals in the United States of America, the availability of a procalcitonin assay result did not lead to a reduction in antibiotic use [43]. The role of procalcitonin in distinguishing between infection and inflammation is less clear—possibly because the distinction between the two is not always in itself completely clear. For example, although gut dysbiosis in acute pancreatitis is not regarded as infection, there are fundamental changes in gastrointestinal permeability which allow bacteria and bacterial products to access the systemic circulation, and there is also activation of intestinal-based inflammatory mediators [44].

The review then examines the current use of procalcitonin measurement in patients with acute pancreatitis.

Procalcitonin as a prognostic tool can be used to assess disease severity and the presence of pancreatic necrosis. Although there is evidence of effectiveness in both these roles, neither is currently recommended for the measurement of procalcitonin. An elevated procalcitonin level, measured on admission to the hospital in patients with acute pancreatitis, indicates a more severe episode, and persistent elevation can be a marker of underlying pancreatic necrosis [45].

However, disease severity in acute pancreatitis should be assessed clinically and reviewed with serial assessment and monitoring supported by CT findings and procalcitonin; simply assessing disease severity is not necessary.

Similarly, as a tool to assess for the presence of pancreatic necrosis, procalcitonin is not the current standard of care. Clinical assessment combined with contrast-enhanced computed tomography is the accepted method.

The principal role of procalcitonin measurement in current practice is to guide antibiotic therapy in patients with acute pancreatitis, where there is a clinical suspicion of infection but in the absence of positive microbiological cultures. In this setting, PROCAP was a large, 260-patient randomized controlled trial using procalcitonin algorithm-based antibiotic prescribing as an intervention in addition to standard care, comparing its effectiveness to standard care alone [10]. The study concluded that procalcitonin-guided care can reduce antibiotic use without increasing infection or harm in patients with acute pancreatitis.

Procalcitonin measurement is not a “magic bullet” and by itself is not the sole answer to antibiotic overuse, but critically, it provides a rational framework for more logical use of antibiotics [46].

## 5. Conclusions

Procalcitonin measurement to guide antibiotic therapy in patients with acute pancreatitis with clinical indications of infection but without positive microbiological cultures is supported by evidence from randomized controlled trials. In patients with acute pancreatitis, where there is concern about possible infection (including raised inflammatory markers) but a lack of positive microbiological cultures, it is appropriate to measure procalcitonin. If the procalcitonin level is >1 µg/L, it is appropriate to prescribe antibiotics. Procalcitonin measurement is a component of modern antibiotic stewardship, which should also include pharmacovigilance with daily review of antibiotic prescribing, microbiological review and frequent cross-sectional imaging.

## Figures and Tables

**Table 1:** Procalcitonin as a predictor of disease severity (prognostic biomarker) in acute pancreatitis.

First Author and Year of Publication	Design	*N*	Sample Collection Time	Cut-Off	Key Findings (For Prediction of Severity)
Rau B et al., 2007 [9].	Prospective multi-centre cohort	104 (predicted severe)	Admission and daily to day 21.	≥3.5 ng/mL on two consecutive days	AUC 0.83 (95% CI, 0.75–0.90) Infected necrosis with MODS.
Liang Y et al., 2019 [27].	Retrospective single-centre	104	Admission	1.80 ng/mL	AUC = 0.906 (0.765–1.050)
Alberti P et al., 2022 [28].	Prospective single-centre	152	For diagnosis of infection	PCT > 0.68 mg/dL	AUC = 0.646 (0.50–0.78) *p* = 0.07
Cho IR et al., 2023 [29].	Retrospective cohort	103	Admission	<0.5 ng/mL	AUC 0.581 (0.470–0.69) *p* = 0.166 Prediction of mild disease.
Yang Q et al., 2024 [30].	Retrospective cohort Severe vs. non-severe	1074	1st 24 h after admission	0.51 ng/mL	AUC 0.7086 (*p* < 0.0001) For prediction of severe disease
Hongli Zhou MM et al., 2025 [31].	Retrospective	300	Admission	Not stated.	AUC 0.85 (0.81–0.89) Sens: 83.6%, Spec 74.9% Youden Index 0.59. *p* < 0.001

**Table 2:** Procalcitonin as a predictor of infected necrosis and episode-related mortality in acute pancreatitis.

First Author and Year of Publication	Design	*N*	Sample Time	Cut-Off	Key Findings
Rau B et al., 2007 [9].	Prospective multi-centre cohort	104 (predicted severe)	Admission and daily to day 21.	≥3.5 ng/mL on two consecutive days	AUC 0.83 (95% CI, 0.75–0.90) Infected necrosis with MODS. AUC 0.91 (95% CI, 0.84–0.96) For mortality.
Choudhuri AH et al., 2020 [34].	Prospective single-centre	42	Admission to ICU and 48 h after		Increase in procalcitonin in non-survivors from 3.8 (1.2–5.6) to 6.2 (1.9–9.1) *p* < 0.01 Mean + sd
Alberti P et al., 2022 [28].	Prospective single-centre	152	For diagnosis of infection	>0.68 ng/mL	AUC = 0.68 (0.53–0.83) *p* = 0.04
Ramirez-Gonzalez LR et al., 2025 [35].	Retrospective single-centre.	59	Admission and at 72 h	1.26 ng/mL at 72 h	Prediction of mortality at 72 h AUC = 0.751 (95% CI: 0.61–0.88) *p* = 0.003
Zhang Y et al., 2025 [36].	Retrospective single-centre	199	Not stated	>1.01 mg/dL in the first 3 days	AUC = 0.716 (0.630–0.801) Sensitivity = 0.638 Specificity = 0.704

**Table 3:** The PROCAP procalcitonin measurement-based algorithm to guide antimicrobial use in patients with acute pancreatitis. In order to use the algorithm, start at the first line of the table and work downwards [10].

**Patient with acute pancreatitis and suspected infection in whom the treating clinician wishes to consider antibiotic therapy or stopping antibiotic therapy (in the absence of positive microbiological cultures)**		
**Suggested management:**	Clinical assessment and measure procalcitonin.	
**Procalcitonin result:**	<1.0 µg/L	>1.0 µg/L
**Recommendation on antibiotic use:**	Do not start antibiotics.; Stop antibiotics in patients already on antibiotic therapy (in the absence of positive microbiology cultures).	Start antibiotics (following local hospital guidelines).
**Follow-up:**	If there is clinical concern about infection: reassess the patient and re-check procalcitonin after 24 h.	
**Repeat procalcitonin result:**	<1.0 µg/L	>1.0 µg/L
**Action on repeat procalcitonin**	No antibiotics.	Continue or start antibiotics (following local hospital guidelines).
**Algorithm over-ride**	Empiric antibiotic use is permitted in patients in whom antibiotics would not be prescribed according to the procalcitonin algorithm but this decision should be made by a senior clinician (attending or consultant), documented and reviewed.	

## Data Availability

There are no data to be made available for this study.

## Abbreviations

The following abbreviations are used in this manuscript:

RR	relative risk
CI	confidence interval
sd	standard deviation of the mean
